# Expert consensus on hospitalization for assessment: a survey in Japan for a new forensic mental health system

**DOI:** 10.1186/1744-859X-10-11

**Published:** 2011-04-08

**Authors:** Akihiro Shiina, Mihisa Fujisaki, Takako Nagata, Yasunori Oda, Masatoshi Suzuki, Masahiro Yoshizawa, Masaomi Iyo, Yoshito Igarashi

**Affiliations:** 1 Department of Psychiatry, Chiba University Hospital, Chiba, Japan; 2 Division of Law and Psychiatry, Chiba University Center for Forensic Mental Health, Chiba, Japan; 3 National Center Hospital of Neurology and Psychiatry, Tokyo, Japan; 4 Chiba Psychiatric Medical Center, Chiba, Japan; 5 Mobara Mental Hospital, Chiba, Japan; 6 Chiba Aoba Municipal Hospital, Chiba, Japan; 7 Department of Psychiatry, Chiba University Graduate School of Medicine, Chiba, Japan

## Abstract

**Background:**

In Japan, hospitalization for the assessment of mentally disordered offenders under the Act on Medical Care and Treatment for the Persons Who Had Caused Serious Cases under the Condition of Insanity (the Medical Treatment and Supervision Act, or the MTS Act) has yet to be standardized.

**Methods:**

We conducted a written survey that included a questionnaire regarding hospitalization for assessment; the questionnaire consisted of 335 options with 9 grades of validity for 60 clinical situations. The survey was mailed to 50 Japanese forensic mental health experts, and 42 responses were received.

**Results:**

An expert consensus was established for 299 of the options. Regarding subjects requiring hospitalization for assessment, no consensus was reached on the indications for electroconvulsive therapy (ECT) or for confronting the offenders regarding their offensive behaviors.

**Conclusions:**

The consensus regarding hospitalization for assessment and its associated problems were clarified. The consensus should be widely publicized among practitioners to ensure better management during the hospitalization of mentally disordered offenders for assessment.

## Background

The need to establish a sophisticated forensic mental health system has increased as a result of the global trend toward the deinstitutionalization of patients with mental disorders [1]. However, for many years, Japan had no specific legal provisions for offenders with mental disorders [2]. Once such offenders were entrusted into the mental health system, they were treated under the Mental Health and Welfare (MHW) Law and were completely detached from the criminal justice system [3].

In 2005, the forensic mental health system in Japan underwent reform along with the enforcement of the Act on Medical Care and Treatment for the Persons Who Had Caused Serious Cases under the Condition of Insanity: the Medical Treatment and Supervision Act (MTS Act) [4]. Under this new system, a person who commits a serious criminal offense while in a state of insanity or with diminished responsibility is be treated and supervised in a judicial administrative frame. The public prosecutor makes allegations to the District Court for the purpose of judgment. The judgment panel consists of one judge and one mental health reviewer ('seishin-hoken-shinpan-in'), with the latter being selected from a group of psychiatrists who hold Judgment Physician license ('seishin-hoken-hantei-i' a national license for forensic mental health specialists). The panel can arrive at three possible verdicts: an order to hospitalize the offender for medical treatment, an order to care for the offender as an outpatient in the community, or a no-treatment order. The offender is then obligated to accept the special psychiatric care supplied by the designated medical facilities and to submit to continuous supervision by a Rehabilitation Coordinator ('shakai-fukki-chousei-kan') working in a probation office.

To return a correct verdict, the MTS Act requires a psychiatric examination. The three essential factors that must be examined when making a treatment order decision are the nature and severity of the mental disorder and its relationship to the offense, the offender's 'treatability' or responsiveness to psychiatric treatment, and the factors that could hinder the person's rehabilitation and the likelihood of a second offense. The offender should be hospitalized for 2 to 3 months during the psychiatric examination, while continuing an appropriate course of psychiatric treatment; this hospitalization period for assessment is known as 'kantei-nyuin' [5].

In 2008, the Japanese Government published a list of 239 Japanese mental hospitals (1.9 per 1,000,000 of the population) for the purpose of hospitalization for assessment of mentally disordered offenders [6]. However, the criteria used to select these facilities are vague.

The MTS Act hardly regulates even the minimum requirements for these facilities. Therefore, remarkable variations exist in the hospitalization conditions for these patients, such as in the availability of human resources, the diagnostic and therapeutic strategies in use, the attitudes regarding ethical issues, and the physical facilities themselves. It had been reported that about 60% to 80% of psychiatrists who treat offenders in designated inpatient facilities find problems with the written reports of psychiatric examinations conducted and written at the assessment stage [7]. In addition, while it is recommended that offenders be treated by a multiple disciplinary team (MDT) similar to that used for regular acute psychiatric care [8], this recommendation was not known at 14% of the facilities that were surveyed [9].

To minimize the variation, and to improve the quality of the assessment, we conducted a written survey that was delivered by mail to leading Japanese forensic mental health experts, and clarified the expert consensus regarding hospitalization for assessment.

## Methods

### Creating the surveys

To create the questionnaire, we formed a working team comprised of judgment physicians, psychiatrists with experience conducting psychiatric examinations, and doctors belonging to facilities for hospitalizations and assessment of mentally disordered offenders. Then, we attempted to extract suitable questionnaire items, which we classified as general introductory questions regarding the characteristics of the facilities (including sections on the 'Structure' and 'Staff') or detailed questions regarding management (including sections on 'Items Before the Start of Examination', 'Diagnosis and Treatment', 'Issues Regarding Informed Consent and Forced Treatment', 'Judgment', and 'Hypothetical Clinical Situations'). We also referred to reviews in the literature to extract questions [7,10]. We then collected the opinions of several experts in an exploratory committee examining 'Research on the Improvement of the System of Hospitalization for Assessment' and revised the questionnaire. Using the above-described procedure, we developed a 60-question survey with 335 options. A sample of the questions is presented in Table 1.

**Table 1:** Sample of the survey questions

Please evaluate the following options for interventions with a subject who refuses to take medication because of a lack of insight into his or her psychiatric disorder, but who is not seriously aggressive			
(1) Explanation and persuasion	1 2 3	4 5 6	7 8 9

(2) Forced medication using liquid or oral disintegrating drugs	1 2 3	4 5 6	7 8 9

(3) Forced intravenous or intramuscular injection	1 2 3	4 5 6	7 8 9

(4) Forced depot injection	1 2 3	4 5 6	7 8 9

(5) Masked medication	1 2 3	4 5 6	7 8 9

(6) Electroconvulsive therapy	1 2 3	4 5 6	7 8 9

(7) Forced medication using a nasal tube	1 2 3	4 5 6	7 8 9

### Rating scale

For the 335 options in the survey, we asked the experts to evaluate the appropriateness of the option using a 9-point scale that was slightly modified from the format developed by the RAND Corporation for ascertaining expert consensus. To develop this rating scale, we referred to the expert consensus guideline series developed by Expert Knowledge Systems, LLC [10]. The anchors of the rating scale are presented in Appendix 1.

### Composition of the expert panel

We identified 50 leading Japanese experts on forensic mental health, focusing on those individuals with extensive experience managing hospitalizations for assessment under the MTS Act. The experts were identified based on their published research in this area and/or their participation in the Japanese Society of Forensic Mental Health or a related association.

### Ethical issues

We reported the contents of this survey to the Ethical Council of the Graduate School of Medicine at Chiba University in advance, and the council declared that the survey did not pose any ethical problems. All the experts were given a written explanation of the purpose of the survey. All respondents provided their informed written consent to participate in the study.

### Data analysis for options scored on the rating scale

For each option, we first defined the presence or absence of a consensus as a distribution unlikely to occur by chance by performing a χ^2 ^test (*P *< 0.05) of the distribution of the scores across three ranges of appropriateness (7-9: appropriate; 4-6: unclear; 1-3: inappropriate). Next, we calculated the mean and 95% confidence interval (CI). A categorical rating of first-line, second-line, or third-line options was designated based on the lowest category in which the CI fell, with boundaries of 6.5 or greater for first-line (preferred) options, 3.5 or greater but less than 6.5 for second-line (alternate) options, and less than 3.5 for third-line (usually inappropriate) options. Among the first line options, we defined an option as 'best recommendation/essential' if at least 50% of the experts rated it as 9. This analysis method was adopted after reference to an expert consensus guideline series [10].

Additionally, we extracted all the items included in the present questionnaire that were also used in a previous questionnaire survey [7] to collect the general opinions of forensic psychiatrists. We then compared the two sets of results to justify the present survey by evaluating the differences between the expert consensus and the general opinion of forensic psychiatrists.

## Results

### Response rate

We received responses from 42 (84%) of the 50 experts to whom the survey was sent. Two of the respondents were female and the rest were male. Of the 42, 2 were professors in the psychiatric department of a university, 14 belonged to a national hospital, 20 belonged to a prefectural hospital, and 6 belonged to a private hospital. All the respondents held a national license as a Designated Physician ('seishin-hoken-shitei-i') under the MHW Law and Judgment Physician in the MTS Act. Furthermore, all the respondents were over 35 years of age and had at least 10 years of experience in psychiatric practice.

All 42 responders answered all the questions adequately. No doubts or criticisms regarding the questionnaire were noted by the experts.

### Degree of consensus

Of the 335 options rated using the 9-point scale, a consensus was reached for 299 (89.3%) options, as defined by the presence of statistical significance using a χ^2 ^test.

**Figure 1:**
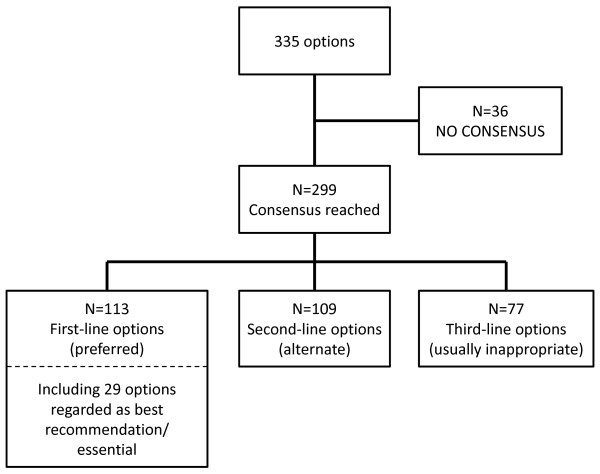
**Degree of consensus**. Of the 335 options rated using the 9-point scale, a consensus was reached for 299 (89.3%) options as defined using a statistically significant χ^2 ^test result.

A total of 113 options were defined as first-line options, of which 29 options were defined as 'best recommendation/essential'. In all, 109 options were defined as second-line options. The remaining 77 options were defined as third-line or usually inappropriate options (see Figure 1).

### Structure

This section consisted of six questions aimed at determining the necessary resources for the appropriate administration of hospitalizations for assessment.

As facilities for the hospitalized assessment of mentally disordered offenders, the best recommendation of the experts was the National Center Hospital, National Center for Neurology and Psychiatry (NCH-NCNP) (mean 8.07; 95% CI 7.62 to 8.53) or an establishment with a specialized facility for the exclusive use of psychiatric examinations (mean 8.03; 95% CI 7.53 to 8.52). For psychiatric examinations, a psychiatric emergency ward (mean 7.61; 95% CI 7.1 to 8.12) or, as a minimum requirement, a psychiatric acute-phase care unit (mean 7.45; 95% CI 6.77 to 7.58) were recommended as first-line options. Medical examination rooms with multiple exit doors (mean 7.64; 95% CI 7.19 to 8.1) were recommended. To prevent self-hanging, a shower without a hose in each bedroom (mean 7.24; 95% CI 6.8 to 7.68) was recommended. As for patient amenities, a television (mean 7.07; 95% CI 6.57 to 7.58) and newspapers (mean 7.26; 95% CI 6.76 to 7.76) were recommended.

### Staff

In this section, we addressed the need for human resources using 16 questions. The participation of Judgment Physicians (mean 8.29; 95% CI 7.93 to 8.54) and Designated Physicians (mean 8.24; 95% CI 8.01 to 8.56) in the hospitalization process was deemed essential. At least 1 staff nurse per 10 inpatients in the assessment ward (mean 7.10; 95% CI 6.56 to 7.64) was recommended. The participation of psychiatric social workers (mean 8.24; 95% CI 7.88 to 8.66) and psychotherapists (mean 8.29; 95% CI 7.87 to 8.7) was also deemed essential. The participation of occupational therapists (mean 7.57; 95% CI 7.07 to 8.07) was recommended. However, a consensus was not reached on whether psychiatric social workers or occupational therapists should be involved in the writing of the examination report.

The formation of an MDT for the psychiatric examination (mean 7.62; 95% CI 7.15 to 8.09) was recommended. However, a consensus was not reached on whether the team should include pharmacists and dietitians or how often the team meetings should be held.

In cases of hospitalization for assessment, the court appoints a case examiner. It was recommended that the examiner not participate in the treatment of the subject directly, but rather that the examiner discusses the treatment strategy with the physician in charge of the subject from time to time (mean 7.17; 95% CI 6.67 to 7.66). In cases where the examiner and the physician in charge disagreed regarding the treatment strategy, the experts did not agree on a first-line option but recommended that the examiner and physician in charge continue their discussion (mean 6.61; 95% CI 5.95 to 7.27). They also recommended that the final decision regarding treatment should be made by the physician in charge (mean 6.56; 95% CI 6.05 to 7.07).

### Items before the start of examination

This section addressed the procedure for accepting offenders to be examined, along with some other institutional issues, and consisted of six questions.

When consulted regarding the acceptance of an offender requiring hospitalization for assessment, the experts did not show any particular first-line options regarding the provision of advance information about the offender. Instead, they preferred to use the offender's category of offense (mean 6.48; 95% CI 5.69 to 7.27) when deciding on either the acceptance or rejection of an offender.

The issue of whether or not medical students should participate in the hospitalization for assessment process did not reach consensus.

### Diagnosis and medical treatment

This section contained questions regarding basic approaches for managing subjects and consisted of six questions.

An interview with the subject (mean 8.55; 95% CI 8.28 to 8.82) and the checking of vital signs (mean 8.74; 95% CI 8.59 to 8.89) on the first day of admission were deemed essential. A family interview (mean 8.55; 95% CI 8.25 to 8.84), consultation with the rehabilitation coordinator in the probation office (mean 8.50; 95% CI 8.22 to 8.78), blood exams (mean 8.81; 95% CI 8.67 to 8.95), intelligence tests (mean 8.43; 95% CI 8.19 to 8.67), personality tests (mean 8.26; 95% CI 7.96 to 8.56) and electroencephalograms (mean 8.21; 95% CI 7.9 to 8.52) performed during the hospitalization period were all deemed as essential. A brain magnetic resonance imaging (MRI) examination (mean 7.40; 95% CI 6.93 to 7.88) was also recommended.

Regarding medication, the prescription of medications to the offenders in the same manner as for other patients with mental disorders (mean 8.24; 95% CI 7.95 to 8.53) was recommended. Regarding psychotherapy, supportive psychotherapy (mean 7.85; 95% CI 7.43 to 8.27) consisting of rapport (mean 7.68; 95% CI 7.16 to 8.2) and psychoeducation (mean 7.22; 95% CI 6.69 to 7.75) were recommended as first-line options.

### Issues regarding informed consent and forced treatment

This section contained eight questions regarding core ethical problems and systematic issues associated with involuntary hospitalization.

The experts recommended that every possible effort to be made to obtain informed consent from the offenders but that the necessary treatment should be enforced upon the patient if consent was not obtained (mean 7.51; 95% CI 7.15 to 7.88). During hospitalization, the need for seclusion or restrictions should be evaluated on a flexible basis (mean 7.52; 95% CI 7 to 8.05), and even if seclusion is decided upon, once the subject has calmed down, the experts recommended that the dayroom be made available to the subjects for a limited time (mean 7.93; 95% CI 7.66 to 8.2) and/or under the observation of the medical staff (mean 7.93; 95% CI 7.62 to 8.24). Seclusion and restriction were to be considered in situations where direct violence to other patients (mean 8.31; 95% CI 8.03 to 8.59), violent behavior or threats of violence towards the staff (mean 7.81; 95% CI 7.49 to 8.13), destroying equipment in the ward (mean 7.81; 95% CI 7.46 to 8.16), clear attempts at suicide (mean 8.19; 95% CI 7.89 to 8.49), or impulsive self-destructive behavior (mean 7.57; 95% CI 7.19 to 7.95) were possibilities.

### Judgment

A panel must judge the acts of the offender and deliver a verdict. This section concerned the judgment process and consisted of four questions.

The experts recommend that the offender's own motivation to recover over the course of hospitalization be carefully evaluated (mean 7.69; 95% CI 7.26 to 8.13). Even after the completion of the psychiatric examination, the continuation of maintenance therapy (mean 8.12; 95% CI 7.69 to 8.55) or therapy to improve his/her mental status (mean 7.05; 95% CI 6.56 to 7.54) until the time of the final judgment was recommended. If the status of the subject changed, leading to a reconsideration of the diagnosis once the results of the psychiatric examination had been reported, a quick report to the panel (mean 8.22; 95% CI 7.87 to 8.53) was essential.

### Hypothetical clinical situations

This section covered several situations that have yet to be adequately addressed in Japan and consisted of 14 questions.

When examining a subject who has committed a homicide, who does not exhibit any obvious psychotic symptoms, and whose past history is unknown, the experts recommend careful observation without medication for a number of days (mean 7.07; 95% CI 6.58 to 7.57).

**Figure 2:**
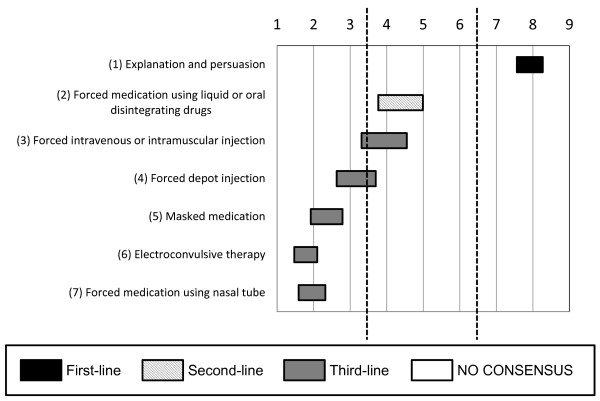
**Options for interventions for subjects who refuse therapy**. With regard to interventions for subjects who refuse to take medication because of a lack of insight into their psychiatric disorder but who are not seriously aggressive, the experts recommend explanation and persuasion.

Regarding the treatment of a subject who refuses to take medication because of a lack of insight into his or her psychiatric disorder, but who is not seriously aggressive (see Figure 2), the experts recommended that only explanation and persuasion be used as treatment options (mean 7.93; 95% CI 7.56 to 8.27).

**Figure 3:**
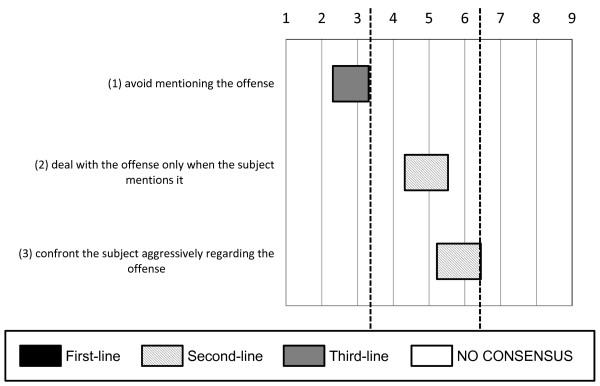
**Options for confronting the subject regarding his or her own offense**. With regard to the confrontation of subjects regarding their offense, the experts did not reach a consensus, but they did not recommend avoiding any mention of the offense.

Regarding the topic of confronting the subject about his or her offense, the experts did not reach a consensus (see Figure 3); they did not recommend avoiding any mention of the offense (mean 2.81; 95% CI 2.31 to 3.31).

**Figure 4:**
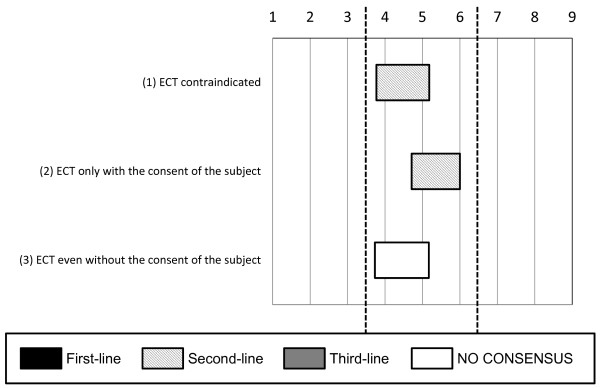
**Options regarding the indications for electroconvulsive therapy (ECT) among subjects who refuse to eat**. The experts did not necessarily approve of the use of electroconvulsive therapy if the subject refused to eat or take drugs because of suicidal thoughts.

**Figure 5:**
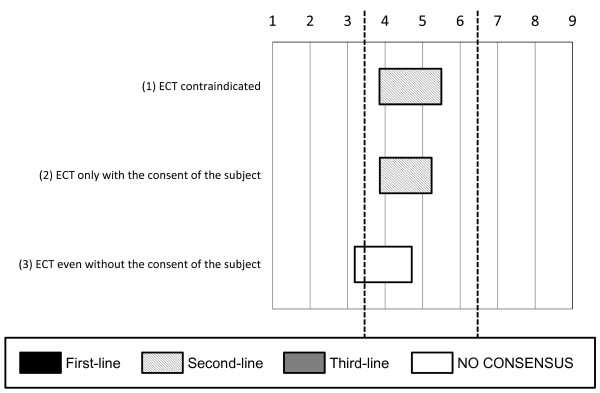
**Options regarding the indications for electroconvulsive therapy (ECT) among subjects with neuroleptic malignant syndrome**. The experts did not necessarily approve of the use of electroconvulsive therapy in subjects with neuroleptic malignant syndrome as a result of previous medications.

The experts did not necessarily approve of the use of electroconvulsive therapy (ECT) if the offender refused to eat or take drugs because of suicidal thoughts (see Figure 4) or after a neuroleptic malignant syndrome caused by previous medications (see Figure 5).

### Comparison of expert consensus and general opinions of forensic psychiatrists

Five items were identified as having the same content as questions included in a past questionnaire survey examining the general opinions of forensic psychiatrists.

In the staff section, regarding the relationship between the case examiner and the physician in charge, 39 of the 105 respondents (37.1%) in the previous survey chose the option 'the case examiner should also be the physician in charge'. This option did not reach a consensus (mean 4.60; 95% CI 3.85 to 5.34) in the present survey. Only 12 of the 42 experts (28.6%) marked this option as being appropriate. However, the percentage of participants who marked this option as being appropriate was not statistically different between the two surveys (Fisher's exact test, *P *= 0.32).

In the diagnosis and medical treatment section, 67 of the 105 respondents (63.8%) in the previous survey chose the option 'to prescribe medications for the offenders in the same way as they would other patients with mental disorders'. As described above, this option reached consensus (mean 8.24; 95% CI 7.95 to 8.53) in the present survey. Of the 42 experts, 40 (95.2%) marked this option as being appropriate. The percentage of participants who marked this option as being appropriate was significantly different between the two surveys (Fisher's exact test, *P *< 0.001).

Regarding issues concerning informed consent and forced treatment, 20 of the 105 respondents (19.0%) in the previous survey chose the option 'continue seclusion (for 1 week or more) even if the offender has calmed down'. This option was not supported by experts (mean 2.14; 95% CI 1.74 to 2.54) in the present survey. None of the experts marked this option as being appropriate. The percentage of participants who marked this option as being appropriate differed significantly between the two surveys (Fisher's exact test, *P *< 0.01).

Regarding hypothetical clinical situations, 33 of the 107 respondents (30.8%) in the previous survey selected the option 'confront the subject regarding his or her offense aggressively'. The experts defined this option as an alternate treatment (mean 5.83; 95% CI 5.22 to 6.45) in the present survey. Of the 42 experts, 16 (38.1%) marked this option as being appropriate. The percentage of participants who marked this option as being appropriate was not significantly different between these two surveys (Fisher's exact test, *P *= 0.40). Regarding the use of ECT, 57 of 105 respondents (54.3%) in the previous survey chose the option 'electroconvulsive therapy should not be performed during the assessment process'. This option did not reach a consensus (mean 5.14; 95% CI 4.34 to 5.95) in the present survey. Of the 42 experts, 16 (38.1%) marked this option as being appropriate. The percentage of participants who marked this option as being appropriate was not significantly different between these two surveys (Fisher's exact test, *P *= 0.08).

The above results are summarized in Table 2.

**Table 2:** Differences between general opinion and expert consensus

Option	Rate of appropriateness		*P *value
		
	General	Experts	
The case examiner should also be the physician in charge	39/105 (37.2%)	12/42 (28.6%)	NS

Medications should be prescribed to offenders in the same manner as they would be for other patients with mental disorders	67/105 (63.8%)	40/42 (95.2%)	<0.001

Continue seclusion (for 1 week or more) even if the offender calms down	20/105 (19.0%)	0/42 (0%)	<0.01

Confront the subject aggressively regarding his or her offense	33/107 (30.8%)	16/42 (38.1%)	NS

Electroconvulsive therapy should not be used during the assessment process	57/105 (54.3%)	16/42 (38.1%)	NS

*P *values were assessed by Fisher's exact test.

NS = not significant.

## Discussion

In the present study, we distributed a written survey to Japanese forensic mental health experts concerning hospitalization for assessment under the new forensic mental health system in Japan. An expert consensus was established for 299 of the 335 options. The results clarified the expert consensus and the current problems associated with the hospitalization for assessment system.

The purpose of hospitalization for the assessment of offenders with mental disorders who have committed serious crimes is to determine the nature and severity of the mental disorder and its relationship to the act, the subject's 'treatability' or responsiveness to psychiatric treatment, and the factors expected to hinder the person's rehabilitation, enabling their best management [4]. Therefore, adequate security is necessary, along with high-quality medical care that includes a well developed infrastructure and staff at the assessment facility.

Regarding infrastructure, the majority of experts named the NCH-NCNP and a specialized facility exclusively dedicated to psychiatric examinations as the best options for use as an assessment facility. Designated medical facilities for inpatient treatment that have been newly established by the MTS act are well equipped for both the security and comfort of the patients. Whether detailed brain imaging systems that are not available at all facilities, such as positron emission tomography or single photon emission computed tomography, are necessary for all offenders subjected to a hospitalization for assessment remains uncertain. Nonetheless, subjects with suspicious organic brain syndromes, including dementia, who exhibit behavioral and psychological symptoms have been reported [11]. Brain-imaging systems may be necessary for the accurate diagnosis of these subjects. Indeed, the experts selected MRI as a first-line option for necessary equipment. A facility that specializes exclusively in psychiatric examinations should be equipped with these machines in addition to the capability of providing adequate security equal to that of a designated medical facility for inpatient treatment.

Regarding staff, the experts claimed that the participation of the Judgment Physicians and the Designated Physicians in the activities at the assessment facility was essential. For appropriate psychiatric examinations, they also recommended that a relatively high nursing staff ratio of 1 nurse for every 10 subjects be adopted. The ratio of 1 nurse per 10 subjects is nearly equal to that in most psychiatric acute-phase care units in Japan and similar to that in the US and Italy but lower than that of specialized forensic psychiatric wards in England, The Netherlands or Japan [12]. Psychiatric social workers and psychotherapists were also deemed essential. The participation of occupational therapists was also recommended. These results suggest that the subjects' behavior, including their interpersonal actions and their responses to medical treatment, should be evaluated by an MDT using intensive psychotherapeutic approaches under minimal seclusions or restrictions. Nonetheless, the majority of facilities performing hospitalizations for assessment are not equipped with psychiatric emergency wards or psychiatric acute-phase care units with access to these necessities [7]. Thus, many psychiatric examinations appear to be performed in inadequate environments, potentially resulting in serious problems.

Regarding psychiatric examinations during the period of hospitalization, family interviews, consultation with Rehabilitation Coordinators in the probation office, typical laboratory medical examinations, intelligence tests, personality tests, and electroencephalograms were deemed essential. Regarding the individuals who should be responsible for writing the examination reports, Judgment Physicians, Designated Physicians and psychotherapists were deemed as being essential to the reporting process, but a consensus was not reached regarding psychiatric social workers or occupational therapists.

Regarding the treatment of the subject during the process, the consensus was that the examiner appointed by the court must not directly treat the subject, but rather should discuss the treatment strategy with the physician in charge of the subject from time to time. If the opinions of the examiner and the physician in charge were conflict regarding the treatment strategy, the experts did not agree on a first-line option, but recommended that the examiner and physician in charge continue their discussion and that the final decision regarding treatment should be made by the physician in charge. The expert consensus indicated the importance of an MDT in the performance of the psychiatric examination. Since there may be a risk of a dual-role dilemma between the evaluator and the therapist if the examiner and the physician in charge are the same person [13], the examiner must not be the physician in charge of the subject [8]. However, this principle is not well known to forensic psychiatrists in Japan. A certain period of time exists between the end of the psychiatric examination and the judge's determination, and the expert consensus is that medical treatment should be continued during this time to maintain or improve the subject's mental status. These results indicate that the hospitalization for assessment system is meant not only to evaluate the offender, but also as a means of therapy.

Regarding the use of ECT, the experts' opinions varied considerably. Ethical issues regarding the use of ECT for forensic patients are often discussed [14], but some forensic subjects with mental disorders actually require ECT. Witzel reported a patient with psychotic depression who was successfully treated using ECT in a forensic psychiatric hospital, supporting the need for ECT in forensic mental health [15]. In Japan, one case report described the use of ECT for a forensic patient after the approval of an ethical council at a designated medical facility for inpatient treatment [16], although ethical councils are not always present at hospitalization for assessment facilities. Another ECT issue is the risk of amnesia, which can be an adverse effect of ECT and may complicate the accurate evaluation of the subject's mental status. At present, the Japanese research group in forensic psychiatry does not recommend the use of ECT, except in very rare situations [17]. Japanese forensic psychiatrists seem to dislike using ECT [7]. The experts also hesitated to use ECT for forensic patients. Overall, the indications for ECT during the hospitalization for assessment process may be limited to life-threatening situations.

The experts recommended that the physician in charge make every possible effort to obtain informed consent from the subject before providing medical treatment but agreed that the necessary treatment should be forced upon the subject if consent cannot be obtained. They also recommended that the need for seclusion or restriction be carefully evaluated and that if a decision to seclude the subject is made, the subject should still be given access to the dayroom for a limited time or while under the observation of the medical staff once he or she has calmed. Above all, the experts recommended that decisions regarding forced treatment during hospitalization for assessment should be made in the same manner as those for general psychiatric treatment. However, this principle has not yet spread among forensic psychiatrists in Japan.

The decision as to whether offenders should be confronted with their own offenses during the hospitalization for assessment process is a complicated one. In forensic settings, psychiatrists often experience the dual-role dilemma of having to evaluate the offender and also act as his or her therapist [13]. In a therapeutic context, it is very important for forensic subjects to reflect on their own past behaviors, and such confrontation methods are effectively used in designated medical facilities in Japan [18,19]. However, such confrontations may create heavy burdens for both the offenders and the medical staff in the context of hospitalizations for assessment. When subjects express their emotions and ideas about their own offenses during the psychiatric examination, it may be important that the medical staff attend to the subjects' confusion and record their emotions without criticism [20].

A limitation of the present research is that the 60 question items included in the survey could not cover all the issues regarding hospitalization for assessment. Although we created the questionnaire used in the present study based on a detailed review and hearing, other problems that we did not consider may be present. Further investigation, possibly including the distribution of a second questionnaire to experts in this field, is needed in the future.

## Conclusions

To the best of our knowledge, this is the first report to survey the attitudes and ideas of forensic mental health experts regarding the hospitalization for assessment process in Japan. The expert consensus about the process is summarized below. The facility should have the necessary infrastructure and human resources to perform adequate psychiatric examinations. MDTs consisting of several specialists should be formed before the start of the psychiatric examination. The examiner and the physician in charge should discuss the treatment strategy of the subject from time to time. Interviews with the subject and his/her family members, physical and mental examinations, and also brain imaging tests should be performed. Medications and non-invasive psychotherapy are recommended just as they are for patients with acute mental disorders. Practitioners should try to obtain informed consent from the patient for all therapies whenever possible and should minimize any seclusions or restrictions. After the completion of the psychiatric examination, therapeutic approaches should continue until the subject has left the facility, and if the status of the subject changes, it should be reported quickly to the court. A consensus was not reached regarding the indications for electroconvulsive therapy and how best to confront the subject with his/her offense during the term of the hospitalization for assessment. The expert consensus differs from the general opinions of forensic psychiatrists in Japan in some aspects. Now that this expert consensus has been reached, it must be widely publicized among practitioners of forensic mental health and fine tuned through critical discussion to enable better management during the hospitalization for assessment process.

## Competing interests

The authors declare that they have no competing interests.

## Authors' contributions

AS, MF, TN, YO, MS and MY conducted the questionnaire. AS conducted the statistical analysis. AS, MF, MI and YI wrote the manuscript. YI acted as the research administrator. All the authors have read and approved the final manuscript.

## Appendix 1

Rating scale

9. The option is extremely appropriate: I always choose to adopt it.

8. The option is usually appropriate: I usually choose to adopt it.

7. The option is usually appropriate: I often choose to adopt it.

6. It is unclear whether the option is appropriate: I choose to adopt it when the situation calls for it.

5. It is unclear whether the option is appropriate: I do not know whether to choose to adopt it.

4. It is unclear whether the option is appropriate: I choose to adopt it only in rare situations.

3. The option is usually inappropriate: I do not adopt it often.

2. The option is usually inappropriate: I seldom adopt it.

1. The option is extremely inappropriate: I never adopt it.

Note: In evaluating each option, first assess which range is most applicable to the option, 'appropriate (points 7-9)' 'inappropriate (points 1-3)' or 'unclear (point 4-6)', and then choose the most applicable point in the chosen range.
